# Periodontal inflammatory disease is associated with the risk of Parkinson’s disease: a population-based retrospective matched-cohort study

**DOI:** 10.7717/peerj.3647

**Published:** 2017-08-10

**Authors:** Chang-Kai Chen, Yung-Tsan Wu, Yu-Chao Chang

**Affiliations:** 1 School of Dentistry, Chung Shan Medical University, Taichung, Taiwan; 2 Section of Dentistry, Zuoying Branch of Kaohsiung Armed Forces General Hospital, Kaohsiung, Taiwan; 3 Department of Physical Medicine and Rehabilitation, Tri-Service General Hospital, School of Medicine, National Defense Medical Center, Taipei, Taiwan; 4 Department of Dentistry, Chung Shan Medical University Hospital, Taichung, Taiwan

**Keywords:** Gingivitis, Parkinson’s disease, Oral health, Periodontitis, Risk factors

## Abstract

**Background:**

The cause−effect relation between periodontal inflammatory disease (PID) and Parkinson’s disease (PD) remains uncertain. The purpose of our study was to investigate the association between PID and PD.

**Methods:**

We conducted a retrospective matched-cohort study by using Taiwan’s National Health Insurance Research Database. We identified 5,396 patients with newly diagnosed PID during 1997–2004 and 10,792 cases without PID by matching sex, age, index of year (occurrence of PID), and comorbidity. Cox proportional hazard regression was used to evaluate the risk of subsequent PD.

**Results:**

At the final follow-up, a total of 176 (3.26%) and 275 (2.55%) individuals developed PD in the case and control groups, respectively. Patients with PID have a higher risk of developing PD (adjusted hazard ratio = 1.431, 95% CI [1.141–1.794], *p* = 0.002).

**Discussion:**

Our results show that PID is associated with an increased risk of developing PD. Whilst these findings suggest that reducing PID may modify the risk of developing PD, further study will be needed.

## Introduction

Parkinson’s disease (PD) is a disabling neurodegenerative disease, which is progressive, and is caused by a loss of dopaminergic neurons in the substantia nigra (Pradeep et al., 2015). Onset is generally after the age of 40 years, and predominantly affects males, with an incidence that increases with age (Van Den Eeden et al., 2003). In Taiwan, the prevalence was 84.8 per 100,000 in 2004, and 147.7 per 100,000 in 2011, with an annual growth rate of 7.9%. The highest prevalence was among individuals over 80 years of age. Over the past decade, Japan, France, and Israel have also reported similar findings (Liu et al., 2016).

Previously, several studies have emphasized inflammatory responses in the progression of PD (Ferrari & Tarelli, 2011; Perry, 2010), and have proposed that chronic conditions and infections, such as diabetes mellitus (Yang et al., 2017) and periodontal problems (Wu et al., 2016), resulting in inflammatory reactions, may be one of the etiological factors in the pathogenesis of PD. In a previous study, after adjustment for age, periodontal disease was significantly higher in men than women (56.4% vs. 38.4%) (Eke et al., 2012). Periodontal inflammatory disease (PID), which comprises two major forms, i.e., chronic gingivitis (CG) and chronic periodontitis (CP), is a form of peripheral inflammation with potentially systemic effects. It involves mechanisms mediated by periodontal pathogenic microbes and inflammatory responses. CG is primarily caused by accumulated dental bacterial plaque and may develop into CP (American Academy of Periodontology–Research et al., 2005). The products of periodontal pathogens cause host cells to generate and release pro-inflammatory mediators, such as IL-1, IL-6, TNF- α, and reactive oxygen species (ROS) (Ebersole & Cappelli, 2000) and the mediators might induce dopaminergic neuronal necrosis or apoptosis, PD initiation and progression, and then cause movement and cognitive disorders (Kaur, Uppoor & Naik, 2016).

Periodontal bacterial cell wall components including Helicobacter pylori (HP), such as the endotoxin lipopolysaccharides (LPS) of Gram-negative strains, are well known as potent inflammatory agents. Bacterial LPS is widely used in model studies of PD induction. In addition, HP infection can aggravate the neurodegenerative process in PD (Kell & Pretorius, 2015; Nielsen et al., 2012; Tan et al., 2015). However, there is no direct evidence to date to indicate that PID plays a role in PD pathogenesis (Kaur, Uppoor & Naik, 2016). Although few articles have addressed the relationship between periodontal problems and PD in cross-sectional studies (Barbe et al., 2017; Cicciu et al., 2012; Muller, Palluch & Jackowski, 2011; Pradeep et al., 2015) the cause-effect relationship remains unclear. In 2013, Liu et al. (2013) first reported an increased risk of parkinsonism after CP in a cohort study. However, only patients with CP were enrolled in their cohort study and a lack of information of CG leaves the exact relationship between PID and PD unclear. Moreover, parkinsonism is a general term that not only indicates PD (Dickson, 2012). Therefore, we here conducted a cohort study using the National Health Insurance Research Database (NHIRD) of Taiwan to determine the risk of developing PD after PID.

## Materials & Methods

### Data sources

The National Health Insurance Program (NHIP) was developed and managed for research purposes since 1995 and provides universal and comprehensive health care for about 99% of Taiwanese residents (Ho Chan, 2010). The NHIRD data from 1996 to 2013 were selected. The data used in the present study were retrieved from the data of one million randomly selected subjects in the whole NHIRD, representing about 4.5% of the population from the entire NHIRD enrollee population (Hsu et al., 2011). There was no significant difference in age and gender between theone million random-sampled data sets and enrollees in the NHIRD. The demographic information gathered included encrypted identification numbers, sex, dates of birth and death, diagnostic data, and procedures. The diagnostic data included the dates of dental procedures and the *International Classification of Diseases*, *Ninth Revision*, *Clinical Modification* (*ICD-9-CM*) diagnostic and procedure codes (Romano, Roos & Jollis, 1993).

The study was approved by the Institutional Review Board (IRB) in Chung Shan Medical University (CS2-15071).

### Study design and sampled participants

This study was a retrospective matched-cohort design. Patients who were aged ≥40 years, new diagnosed between January 1, 1997, and December 31, 2004, based on the ICD-9-CM diagnostic criteria code: 523.1 (CG) and 523.4 (CP), were recruited. In addition, each enrolled patient had been diagnosed at least at three outpatient clinics with PID (CG or/and CP) during a 1-year study period (Tzeng et al., 2016). Exclusion criteria were as follows: age and gender unknown, and PID diagnoses made before 1997. In addition, the patients being diagnosed with PD (ICD-9-CM code: 332.0) (Liu et al., 2016) before 1997 or before the first visit for PID were also excluded. In the interests of accuracy patients were excluded if they had not accessed health services for more than one year, as the NHIRD does not record deaths. A total of 5,396 patients with PID were recruited and 10,792 patients without PID were matched by gender, age, and index years as a control group, at a 1:2 ratio.

**Figure 1:**
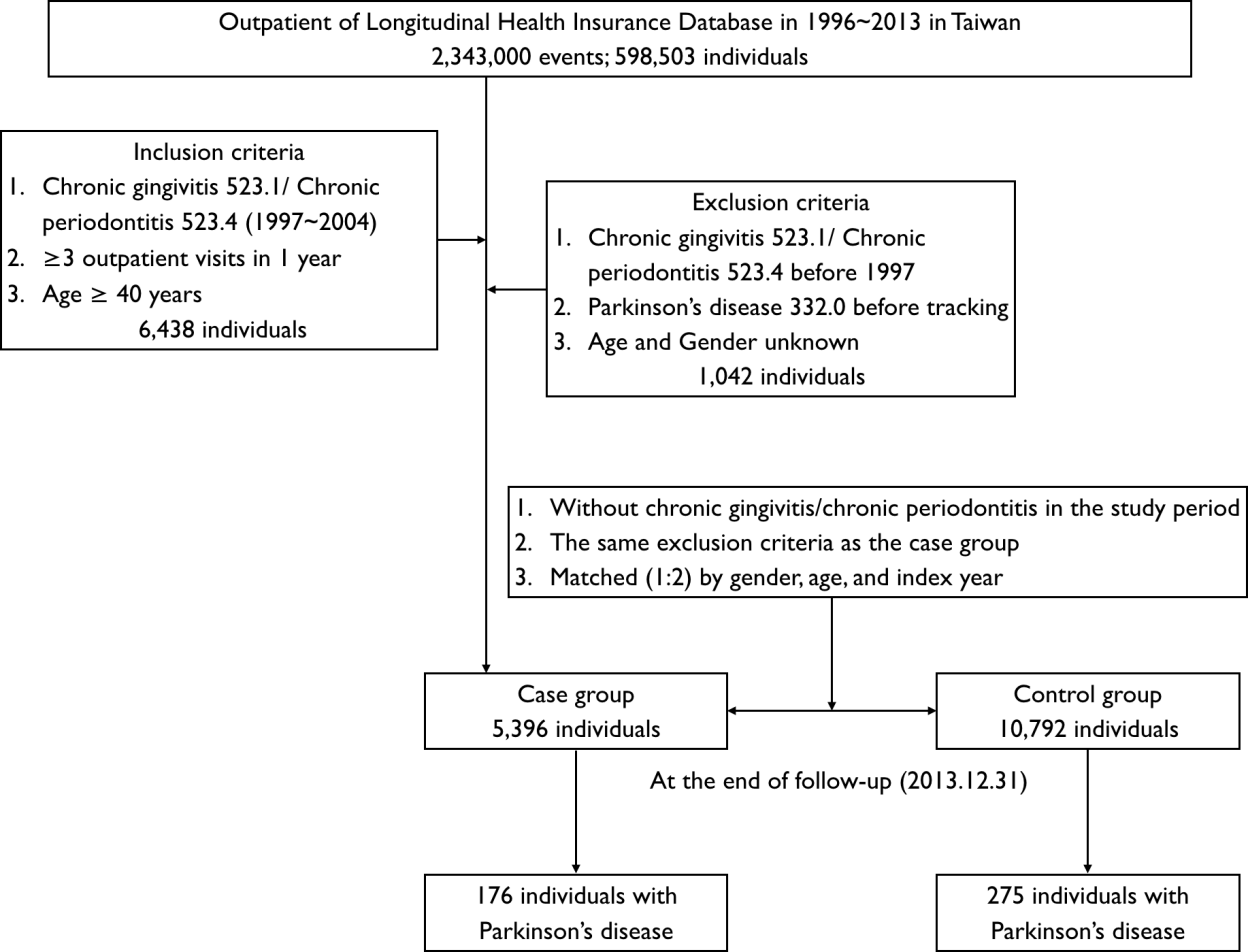
Flowchart of cohort selection of patients from the National Health Research Institute.

Both cohorts were followed from the index date until the PD diagnosis, death, or the end of December 31, 2013, whichever came first, as shown in Fig. 1. The covariates included gender and age group (40–49, 50–59, 60–69, and ≥70 years). According to the definition of urbanization issued by the National Institutes of Health in Taiwan, all 365 townships in Taiwan are divided into seven clusters according to the following variables: population density (people/km^2^), the proportion of the population with college or above educational levels, population ratio of elderly people (over 65 years old), the population ratio of people who are agricultural workers and the number of physicians per 100,000 people. In the present study, we operationally defined townships of 1–2 clusters as level 1, 3–4 clusters as level 2, and 5–7 clusters as level 3 (Liu et al., 2006).

The PD-related comorbidities include hypertension (ICD-9-CM codes: 401.1, 401.9, 402.10, 402.90, 404.10, 404.90, 405.1, and 405.9), hyperlipidemia (ICD-9-CM codes: 272.0–272.9), chronic kidney disease (ICD-9-CM codes: 580, 581–589, 753, 403, 404, 250.4, 274.1, 440.1, 442.1, 447.3, 572.4, 642.1, and 646.2), depression (ICD-9-CM code: 311), stroke (ICD-9-CM codes: 433, 434, and 436) and traumatic brain injury (ICD-9-CM codes: 800–804, 850–854, 905.0, 950.1, 950.3, 907.0, 959.01, 959.9, 310.2, and V15.52) (Chang et al., 2016; Goodarzi et al., 2016; Hsu et al., 2016; Liu et al., 2013; Wu et al., 2016). We also recorded the Charlson Comorbidity Index (CCI), which contains 17 weighted comorbidities and was calculated for each participant (Charlson et al., 1987).

### Statistical analysis

The *t*-test and chi-square test were used to compare the demographic and clinical characteristics of patients with PD vs. those without PID. Univariate and multivariate models were then used to calculate the hazard ratio (HR) and the 95% confidence interval (CI) with stratified Cox regression models. Multivariable models were adjusted for PD-related comorbidities, CCI score, and urbanization level. The incidence rate (IR) and incidence rate ratio (IRR) (per 100,000 person-years) was calculated by dividing the number of events of current PD by the person-years (PYs) observed for each patient. The Kaplan–Meier method was used to assess the survival probability in PD between the case and control cohorts. The log-rank test was used to compare their differences.

In sensitivity analysis, we identified patients with PID that occurred ≥1-year after the diagnosis of PID and the incidence of PD during the 5-year period after a diagnosis of CG and CP (Liu et al., 2013). We performed sensitivity analysis, excluding patients diagnosed with PD < 1–5 years after a diagnosis of CG or CP, to ensure the stability and accuracy of the statistical model (Wong et al., 2016). All statistical analyses were performed in SAS version 9.3 (SAS Institute, Cary, NC, USA) and SPSS software version 22 (SPSS Inc., Chicago, IL, USA). Statistical significance was defined by a *p*-value <0.05.

## Results

The baseline demographic characteristics at the beginning of the study were shown in Table 1. The patients with PID had a higher prevalence of hyperlipidemia, depression, CCI score, and urbanization level than the control cohort. The mean ages for the case and control cohorts were 54.1 ± 10.5 and 54.2 ± 10.5 years, respectively. The mean follow-up time for the case and control cohorts was 11.9 ± 2.6 and 12.2 ± 2.6 years, respectively.

**Table 1:** Demographic characteristics of the study cohort at baseline.

Variable	Total		Periodontal inflammatory disease				*p*-value
			With (case)		Without (control)		
	*n*	%	*n*	%	*n*	%	
Total	16,188	100	5,396	100	10,792	100	
Gender							
Female	7,461	46.09	2,487	46.09	4,974	46.09	>0.999
Male	8,727	53.91	2,909	53.91	5,818	53.91	
Age (years)							
40–49	6,808	42.06	2,269	42.05	4,539	42.06	>0.999
50–59	4,610	28.48	1,537	28.48	3,073	28.47	
60–69	2,939	18.16	980	18.16	1,959	18.15	
≥70	1,831	11.31	610	11.3	1,221	11.31	
Hypertension							
No	7,461	46.09	2,487	46.09	4,974	46.09	0.1834
Yes	8,727	53.91	2,909	53.91	5,818	53.91	
Hyperlipidemia							
No	13,015	80.4	4,198	77.8	8,817	81.7	<0.0001
Yes	3,173	19.6	1,198	22.2	1,975	18.3	
Chronic kidney disease							
No	11,256	69.53	3,727	69.07	7,529	69.76	0.3651
Yes	4,932	30.47	1,669	30.93	3,263	30.24	
Depression							
No	13,829	85.43	4,522	83.8	9,307	86.24	<0.0001
Yes	2,359	14.57	874	16.2	1,485	13.76	
Stroke							
No	13,434	82.99	4,478	82.99	8,956	82.99	>0.999
Yes	2,754	17.01	918	17.01	1,836	17.01	
Traumatic brain injury							
No	13,517	83.5	4,545	84.23	8,972	83.14	0.0773
Yes	2,671	16.5	851	15.77	1,820	16.86	
CCI score							
0	1,719	10.62	541	10.03	1,178	10.92	0.0010
1	2,455	15.17	747	13.84	1,708	15.83	
2	2,583	15.96	872	16.16	1,711	15.85	
≥3	9,431	58.26	3,236	59.97	6,195	57.4	
Urbanization level							
1	10,479	64.73	6,662	61.71	3,817	70.79	<0.0001
2	4,337	26.79	3,076	28.49	1,261	23.39	
3	1,372	8.48	1,058	9.8	314	5.82	

**Notes.**

CCI charlson Comorbidity Index

A total of 176 (3.26%) and 275 (2.55%) patients were diagnosed with PD in the case and control cohorts, respectively (Fig. 1). Table 2 shows the Cox regression analysis of risk factors associated with development of PD. More people developed PD in the PID cohort than in the control cohort and the adjusted HR was 1.431 (95% CI [1.141–1.794], *p* = 0.002; Table 2). Patients with hypertension, depression, stroke, traumatic brain injury and CCI score ≥3 tended to have a higher risk of development of PD and the adjusted HR was 1.746, 2.116, 2.257, 1.645 and 4.207 respectively (all *p* < 0.05; Table 2).

**Table 2:** Covariates associated with Parkinson’s disease at the end of follow-up with univariate and multivariable Cox-regression analysis.

Variable	Univariate analysis			Multivariable analysis		
	Crude HR	95% CI	*p*-value	Adjusted HR	95% CI	*p*-value
Periodontal inflammatory disease						
Control	Reference			Reference		
Case	1.422	1.165–1.737	0.0005	1.431	1.141–1.794	0.002
Hypertension						
No	Reference			Reference		
Yes	2.566	1.727–3.813	<0.0001	1.746	1.114–2.735	0.015
Hyperlipidemia						
No	Reference			Reference		
Yes	2.566	1.727–3.813	<0.0001	1.018	0.763–1.36	0.9019
Chronic kidney disease						
No	Reference			Reference		
Yes	1.358	1.063–1.735	0.0144	0.992	0.746–1.32	0.9586
Depression						
No	Reference			Reference		
Yes	2.654	1.995–3.53	<0.0001	2.116	1.55–2.888	<0.0001
Stroke						
No	Reference			Reference		
Yes	2.981	2.289–3.881	<0.0001	2.257	1.693–3.007	<0.0001
Traumatic brain injury						
No	Reference			Reference		
Yes	2.035	1.548–2.676	<0.0001	1.645	1.217–2.224	0.0012
CCI score						
0	Reference			Reference		
1	3.778	0.979–14.584	0.0538	3.433	0.85–13.873	0.0834
2	4.202	1.156–15.282	0.0293	2.829	0.753–10.635	0.1238
≥3	9.472	2.821–31.808	0.0003	4.207	1.171–15.107	0.0276
Urbanization level						
1	Reference			Reference		
2	1.288	0.977–1.696	0.0722	1.230	0.905–1.671	0.1862
3	1.187	0.804–1.752	0.3891	1.133	0.713–1.799	0.5981

**Notes.**

CCI Charlson Comorbidity Index HR hazard ratio CI confidence interval

Table 3 shows subgroups stratified by gender, age, comorbidities, CCI score and urbanization level during a 1-year period. The IRR of PD was significantly higher among the case cohort than it was among the control cohort, in the following subgroups: male gender, age ≥60 years, hypertension, stroke, CCI score 1, and CCI score ≥3. Both the patients with and without hyperlipidemia, chronic kidney disease, and traumatic brain injury in the case group were at higher risk of PD than were the control group. Level 1 and level 2 carried greater significant risk than did level 3 in terms of urbanization. However, PID subjects who were male, aged ≥70 years, hypertension, no hyperlipidemia, no depression, stroke, with/without chronic kidney disease, traumatic brain injury, CCI score ≥3, and the highest urbanization level 1, were associated with significant higher risk of PD after adjusting the HR.

**Table 3:** Incidence and hazard ratios of Parkinson’s disease with periodontal inflammatory disease at the end of follow-up period, stratified by variables listed in the table with Cox-regression analysis.

Variable	Periodontal inflammatory disease						IRR	95% CI	Adjusted HR	95% CI	*p*-value
	With (case)			Without (control)							
	Event	PYs	IR	Event	PYs	IR					
Gender											
Female	62	29,547	209.8	119	60,748	195.9	1.07	0.88–1.30	1.145	0.839–1.564	0.3932
Male	114	34,501	330.4	156	70,786	220.4	1.50^*^	1.26–1.78	1.557^*^	1.216–1.993	0.0004
Age (years)											
40–49	5	27,514	18.2	18	56,114	32.1	0.57	0.32–1.01	0.716	0.264–1.946	0.5129
50–59	26	18,099	143.7	48	37,100	129.4	1.11	0.88–1.41	1.414	0.867–2.306	0.1657
60–69	64	11,575	552.9	107	23,884	448.0	1.23^*^	1.09–1.39	1.204	0.878–1.651	0.2483
≥70	81	6,860	1180.8	102	14,437	706.5	1.67^*^	1.52–1.83	1.615^*^	1.198–2.177	0.0017
Hypertension											
No	17	23,513	72.3	24	46,242	51.9	1.39	0.97–1.99	1.563	0.815–2.997	0.1785
Yes	159	40,534	392.3	251	85,292	294.3	1.33^*^	1.14–1.55	1.365^*^	1.115–1.671	0.0026
Hyperlipidemia											
No	133	49,693	267.6	214	10,7219	199.6	1.34^*^	1.12–1.61	1.471^*^	1.180–1.833	0.0006
Yes	43	14,354	299.6	61	24,315	250.9	1.19^*^	1.01–1.41	1.097	0.739–1.629	0.6457
Chronic kidney disease											
No	86	44,191	194.6	141	91,486	154.1	1.26^*^	1.02–1.56	1.363^*^	1.037–1.793	0.0266
Yes	90	19,857	453.2	134	40,049	334.6	1.35^*^	1.17–1.55	1.370^*^	1.044–1.797	0.0232
Depression											
No	113	53,832	209.9	184	11,3417	162.2	1.29^*^	1.05–1.58	1.408^*^	1.108–1.788	0.0050
Yes	63	10,216	616.7	91	18,117	502.3	1.23^*^	1.09–1.38	1.313	0.946–1.822	0.1038
Stroke											
No	70	53,310	131.3	123	10,9094	112.7	1.16	0.90–1.49	1.209	0.896–1.633	0.2144
Yes	106	10,737	987.2	152	22,440	677.4	1.46^*^	1.32–1.61	1.479^*^	1.149–1.904	0.0024
Traumatic brain injury											
No	112	54,111	207.0	186	10,9259	170.2	1.22^*^	1.00–1.49	1.213	0.956–1.539	0.1121
Yes	64	9,936	644.1	89	22,276	399.5	1.61^*^	1.42–1.82	1.690^*^	1.215–2.350	0.0018
CCI score											
0	1	6,346	15.8	2	14,132	14.2	1.11	0.54–2.27	1.495	0.025–88.223	0.8468
1	6	8,767	68.4	8	20,399	39.2	1.75^*^	1.18–2.59	2.139	0.711–6.439	0.1762
2	9	10,343	87.0	17	20,698	82.1	1.06	0.78–1.43	1.463	0.636–3.365	0.3706
≥3	160	38,591	414.6	248	76,305	325.0	1.28^*^	1.11–1.48	1.328^*^	1.084–1.626	0.0061
Urbanization level											
1	124	45,606	271.9	144	81,426	176.8	1.54^*^	1.27–1.86	1.467^*^	1.151–1.871	0.0020
2	44	14,747	298.4	84	37,197	225.8	1.32^*^	1.11–1.57	1.426	0.986–2.062	0.0593
3	8	3,695	216.5	47	12,911	364.0	0.59^*^	0.50–0.70	0.581	0.272–1.241	0.1606

**Notes.**

* *p* < 0.05.

CCI Charlson Comorbidity Index PYs person-years IR incidence rate (per 10^5^ PYs) IRR incidence rate ratio (per 10^5^ PYs) CI confidence interval HR hazard ratio

Applying sensitivity analysis to the strategic evaluation by using the Cox proportional hazards regression model for examining the risk of PD after CG and CP were shown in Table 4. We performed sensitivity analysis after excluding patients diagnosed with PD < 1 and <5 years after diagnosis of CG and CP. The association between CG/CP and PD remained consistent (adjusted HR of 1-year and 5-year was 1.431 and 1.395 respectively.)

**Figure 2:**
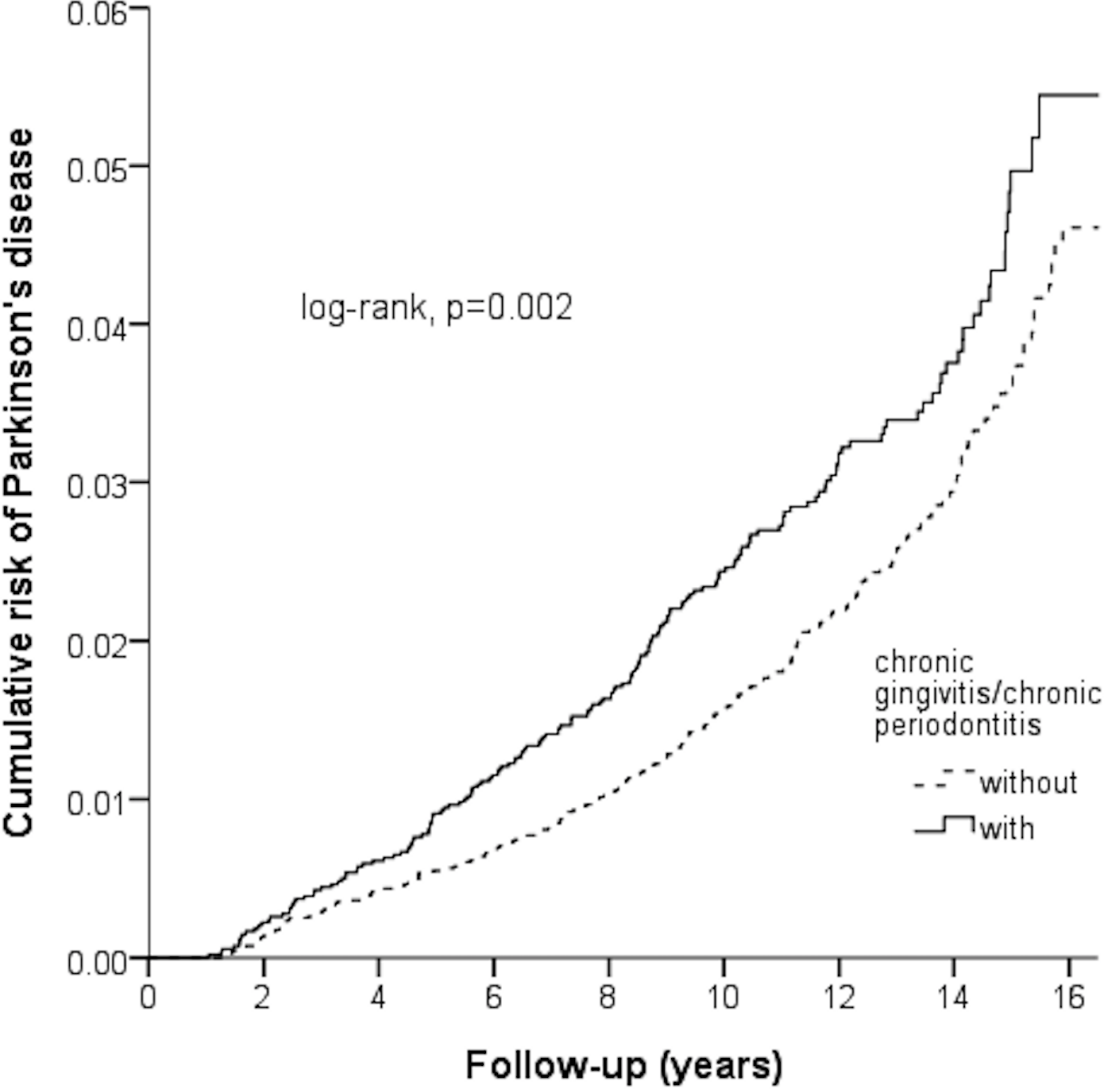
Kaplan–Meier model based on the Cox-regression analysis for the cumulative risk of Parkinson’s disease among the case and control cohort with the log-rank test.

Figure 2 shown the Kaplan–Meier for cumulative risk of PD in the case and control groups. The difference between the case and control groups reached statistical significance difference between the case and control group in the 1st year of follow-up (*p* < 0.05 with log-rank test).

## Discussion

To the best of our knowledge, this is the first nationwide population-based matched-cohort study to find that patients with newly diagnosed PID had an increased risk of developing PD (adjusted HR = 1.431) regardless of comorbidities, CCI score, and urbanization level. Overall, our study found that hypertension, depression, stroke, traumatic brain injury, and CCI score ≥3 were independent risk factors for PD.

**Table 4:** Sensitivity analysis of Cox-regression model for Parkinson’s disease.

Periodontal inflammatory disease	Crude HR	95% CI	*p*-value	Adjusted HR	95% CI	*p*-value
1-year period after a diagnosis	1.422	1.165–1.737	0.0005	1.431	1.141–1.794	0.002
5-year period after a diagnosis	1.364	1.079–1.725	0.0095	1.395	1.067–1.825	0.015

**Notes.**

HR hazard ratio CI confidence interval

Periodontal microorganisms mainly comprise the gram-negative bacteria with endotoxin LPS, which leads to breakdown of the blood brain barrier (BBB). PID can lead to the induction of pro-inflammatory cytokines, including IL-1, IL-6, and TNF-α. These cytokines can activate microglial cells, which produce nitric oxide and ROS, leading to death of dopaminergic neurons (Kaur, Uppoor & Naik, 2016). Our findings showing increased PD after PID support the reports stating that LPS, produced by gram-negative bacteria, may be an important contributor to the development and progression of PD (Bohatschek, Werner & Raivich, 2001). Similar findings were reported from several studies, where the correlation between periodontal problems and PD were revealed. They reported that periodontal problems are frequently found in patients with PD by using cross-section observation, because these patients with PD progressively showed less self-care capability and reduced dental appointments (Barbe et al., 2017; Cicciu et al., 2012; Muller, Palluch & Jackowski, 2011; Pradeep et al., 2015; Schwarz, Heimhilger & Storch, 2006). In addition, a previous study found that patients with PD frequently have oral gram-negative bacteria. Their oral flora differed considerably from that of healthy people (Gosney et al., 2003). Moreover, patients with PD often suffer from xerostomia, drooling, and dysphagia, which impair their quality of life (Barbe et al., 2017). Therefore, periodontal disease is also one of the important issues affecting the quality of life in patients with PD (Cicciu et al., 2012). Nonetheless the cause–effect relationship between periodontal problems and PD remained uncertain until Liu et al. (2013) reported, using a cohort study, that revealed increased Parkinsonism five years following a diagnosis.

Unfortunately, patients with CG were not enrolled and the definite HR for a time-period less than 5 years after diagnosis of PID was not reported in their study. We emphasize the importance of enrolling CG because gingivitis is caused by accumulated dental-bacterial plaque and inflammation of gingiva, and CP may develop in PID. CG does not directly result in tooth loss and may be considered reversible with appropriate care. However, CP is always preceded by CG and any resulting clinical loss of attachment and alveolar bone loss due to CP cannot be reversed. In our study, we found that patients with PID (mainly CG and CP) had an almost 1.4-fold increased risk of developing PD, not only in the CP stage. Therefore, greater care should be paid, to educating the oral hygiene and plaque control methods for patients with PD because periodontal pathology presented a high prevalence in the early stages of gingivitis.

As shown in Table 3, we found male gender, age ≥70 years, hypertension, stroke, traumatic brain injury, CCI score ≥3, and urbanization level 1 in patients with PID were significantly associated with the risk of developing PD. Our findings agree with recent studies which found that PD incidence was higher among males than females (Smith & Dahodwala, 2014). The possible explanations suggested that women carry recessive susceptibility genes on the X chromosome, estrogen has neuroprotective effects, women have a lower rate of toxic exposure, and less incidence of traumatic brain injury, than men (Smith & Dahodwala, 2014). The patients with and without chronic kidney disease were related with the increased risk of development in PD (Linnemann Jr & First, 1979).

PID is a chronic inflammatory condition of the supporting structures of the teeth resulting from a dental plaque biofilm attached to teeth surfaces. Previous study has indicated the presence of a significant association between periodontitis and hyperlipidemia (Cutler et al., 1999). According to an earlier study (Chung et al., 2014), we adjusted for the selected comorbidities including hyperlipidemia in the Cox-regression model. However, in the subgroups stratified by gender, age, comorbidities, CCI score and urbanization (Table 3), the patients without hyperlipidemia were associated with a higher risk of developing PD (adjusted HR = 1.471). Therefore, further investigation will be required to confirm and clarify the mechanism.

Our results showed higher IRR and adjusted HR for urbanization level 1 (1.54 and 1.467, respectively) in the case group for developing PD. It may be explained by the urban–rural differences in terms of lifestyle, availability of medical resources, and convenience of medical access due to urban patients having better health care (Liu et al., 2016). We performed sensitivity analysis to evaluate the role of PID in the development of PD. We further demonstrated and confirmed the adjusted HR during the 1-year and 5-year follow-up period for individuals with PID were 1.431 and 1.395 relative to the control group. However, in a previous study, the patients exposed to CP had significantly greater adjusted HR than did the control group after the 5-year follow-up period (Liu et al., 2013). Based on our findings, we suggest that it is necessary to control inflammatory components of patients in the early phase, to potentially reduce the risk of PD.

Our research has the following advantages: (1) We applied a nationwide database and recruited a large number of sample sizes in highlighting the HR and IR over the 16-year long-term follow-up. (2) Taiwanese NHIRD provides continued coverage for the whole population of Taiwan and thus avoids selection bias in the cohorts, (3) the use of the NHIRD eliminates the need to minimize patients in the cohort that were lost to tracing, (4) in socio-demographic characteristics, it is easy to obtain geographically dispersed large samples, which avoids the estimated regional discrepancy (Liu et al., 2006), (5) we applied a rigorous definition to identify patients with PD (ICD-9-CM code: 332.0), such that statistical analysis would be more robust and reliable.

However, there were some limitations to our study (1) We excluded patients who had PD before tracing. However, we could not differentiate between primary and secondary Parkinsonism in analyzing NHIRD on the diagnostic code from a representative cohort (Liu et al., 2016), (2) we did not access medical records of all defined PID and PD cases, because all the medical records from the NHIRD was de-identified due to ethics approval. We had no clinical information regarding image findings, clinical photographs and examinations of the periodontal disease, laboratory data or treatment response in the defined patients. (3) Periodontal treatment in clinics, oral hygiene from caregivers improved education regarding good oral hygiene practices may help to prevent PD by reducing inflammation (Pradeep et al., 2015); however, personal details about periodontal therapy were not included in the NHIRD. (4) Finally, our methods to extract data from the NIHRD enable long-term follow-up periods of sufficiently large cohorts to correlate risk for PD in the context of PID and in the future, could incorporate additional factors such as environmental exposures, lifestyle (e.g., smoking) and genetic polymorphisms. Accurate risk assessment for PD in the context of PID is necessary if it is to influence healthcare planning and national health insurance policy.

## Conclusions

Individuals exposed to PID were 1.431 times more likely to develop PD than those who were not exposed. However, future long-term, larger or national data sets combined with genes, environmental exposure, lifestyle changes, dietary habits, and accurately defined PD diagnosis should be investigated to support the current research results.

##  Supplemental Information

10.7717/peerj.3647/supp-1 Supplemental Information 1 Analysis of PD raw data

## Additional Information and Declarations

### Data Availability

The following information was supplied regarding data availability: The raw data has been deposited at the National Health Insurance Research Database in LHID 2010, and has been uploaded as Supplemental Information 1.
