# Supplementary material for: Periodontal inflammatory disease is associated with the risk of Parkinson’s disease: a population-based retrospective matched-cohort study
Source: PeerJ. 2017 Aug 10;5:e3647. doi: 10.7717/peerj.3647 (PMC5554596; doi:10.7717/peerj.3647)

periodontal code :523.1, 523.4
data collection in 1997-2004
3 clinical visitis

variation		Total		Chronic Periodontitis				
				No		Yes		
		n	%	n	%	n	%	p
age	40-49	6808	42.06	4539	42.06	2269	42.05	>0.999
	50-59	4610	28.48	3073	28.47	1537	28.48	
	60-69	2939	18.16	1959	18.15	980	18.16	
	70+	1831	11.31	1221	11.31	610	11.3	
	Total	16188	100	10792	100	5396	100	
gender	F	7461	46.09	4974	46.09	2487	46.09	>0.999
	M	8727	53.91	5818	53.91	2909	53.91	
Hypertension	No	7461	46.09	4974	46.09	2487	46.09	0.1834
	Yes	8727	53.91	5818	53.91	2909	53.91	
Hyperlipidemia	No	13015	80.4	8817	81.7	4198	77.8	<0.0001
	Yes	3173	19.6	1975	18.3	1198	22.2	
Chronic kidney disease	No	11256	69.53	7529	69.76	3727	69.07	0.3651
	Yes	4932	30.47	3263	30.24	1669	30.93	
Depression	No	13829	85.43	9307	86.24	4522	83.8	<0.0001
	Yes	2359	14.57	1485	13.76	874	16.2	
Stroke	No	13434	82.99	8956	82.99	4478	82.99	>0.999
	Yes	2754	17.01	1836	17.01	918	17.01	
Traumatic brain injury	No	13517	83.5	8972	83.14	4545	84.23	0.0773
	Yes	2671	16.5	1820	16.86	851	15.77	
CCI	0	1719	10.62	1178	10.92	541	10.03	0.0010
	1	2455	15.17	1708	15.83	747	13.84	
	2	2583	15.96	1711	15.85	872	16.16	
	>=3	9431	58.26	6195	57.4	3236	59.97	
Urbanizaiton level	level 1	10479	64.73	3817	70.79	6662	61.71	<0.0001
	level 2	4337	26.79	1261	23.39	3076	28.49	
	level 3	1372	8.48	314	5.82	1058	9.8	
Parkinson's Dissease	No	15737	97.21	10517	97.45	5220	96.74	0.0093
	Yes	451	2.79	275	2.55	176	3.26	

incidence

variation		Chronic Periodontitis													
		No			Yes										
		n	PYs	ID0	n	PYs	ID1	IDR	95%CI-LL	95%CI-UL	sig	Adjuted HR	95%CI-LL	95%CI UL	p
age	40-49	18	56114	32.1	5	27514	18.2	0.57	0.32	1.01		0.716	0.264	1.946	0.5129
	50-59	48	37100	129.4	26	18099	143.7	1.11	0.88	1.41		1.414	0.867	2.306	0.1657
	60-69	107	23884	448.0	64	11575	552.9	1.23	1.09	1.39	+	1.204	0.878	1.651	0.2483
	70+	102	14437	706.5	81	6860	1180.8	1.67	1.52	1.83	+	1.615	1.198	2.177	0.0017
	Total	275	131534	209.1	176	64048	274.8	1.31	1.09	1.57	+	1.364	1.125	1.654	0.0016
gender	F	119	60748	195.9	62	29547	209.8	1.07	0.88	1.30		1.145	0.839	1.564	0.3932
	M	156	70786	220.4	114	34501	330.4	1.50	1.26	1.78	+	1.557	1.216	1.993	0.0004
Hypertension	No	24	46242	51.9	17	23513	72.3	1.39	0.97	1.99		1.563	0.815	2.997	0.1785
	Yes	251	85292	294.3	159	40534	392.3	1.33	1.14	1.55	+	1.365	1.115	1.671	0.0026
Hyperlipidemia	No	214	107219	199.6	133	49693	267.6	1.34	1.12	1.61	+	1.471	1.180	1.833	0.0006
	Yes	61	24315	250.9	43	14354	299.6	1.19	1.01	1.41	+	1.097	0.739	1.629	0.6457
Chronic kidney disease	No	141	91486	154.1	86	44191	194.6	1.26	1.02	1.56	+	1.363	1.037	1.793	0.0266
	Yes	134	40049	334.6	90	19857	453.2	1.35	1.17	1.55	+	1.370	1.044	1.797	0.0232
Depression	No	184	113417	162.2	113	53832	209.9	1.29	1.05	1.58	+	1.408	1.108	1.788	0.0050
	Yes	91	18117	502.3	63	10216	616.7	1.23	1.09	1.38	+	1.313	0.946	1.822	0.1038
Stroke	No	123	109094	112.7	70	53310	131.3	1.16	0.90	1.49		1.209	0.896	1.633	0.2144
	Yes	152	22440	677.4	106	10737	987.2	1.46	1.32	1.61	+	1.479	1.149	1.904	0.0024
Traumatic brain injury	No	186	109259	170.2	112	54111	207.0	1.22	1.00	1.49	+	1.213	0.956	1.539	0.1121
	Yes	89	22276	399.5	64	9936	644.1	1.61	1.42	1.82	+	1.690	1.215	2.350	0.0018
Urbanizaiton level	level 1	144	81426	176.8	124	45606	271.9	1.54	1.27	1.86	+	1.467	1.151	1.871	0.0020
	level 2	84	37197	225.8	44	14747	298.4	1.32	1.11	1.57	+	1.426	0.986	2.062	0.0593
	level 3	47	12911	364.0	8	3695	216.5	0.59	0.50	0.70	-	0.581	0.272	1.241	0.1606
CCI	0	2	14132	14.2	1	6346	15.8	1.11	0.54	2.27		1.495	0.025	88.223	0.8468
	1	8	20399	39.2	6	8767	68.4	1.75	1.18	2.59	+	2.139	0.711	6.439	0.1762
	2	17	20698	82.1	9	10343	87.0	1.06	0.78	1.43		1.463	0.636	3.365	0.3706
	>=3	248	76305	325.0	160	38591	414.6	1.28	1.11	1.48	+	1.328	1.084	1.626	0.0061

ID： Incidence Density , PYs ×100,000
IDR=ID1/ID0
sig :significance

Adjusted HR

Uni-Cox-regression

Parameter	DF	Estimate	StdErr	ChiSq	ProbChiSq	HazardRatio	HRLowerCL	HRUpperCL
group	1	0.35232	0.10185	11.9668	0.0005	1.422	1.165	1.737
Hypertension	1	0.94235	0.20204	21.7541	<.0001	2.566	1.727	3.813
Hyperlipidemia	1	0.94235	0.20204	21.7541	<.0001	2.566	1.727	3.813
Chronic kidney disease	1	0.30596	0.12507	5.9849	0.0144	1.358	1.063	1.735
Depression	1	0.97595	0.14565	44.8995	<.0001	2.654	1.995	3.53
Stroke	1	1.09213	0.13462	65.8142	<.0001	2.981	2.289	3.881
Traumatic brain injury	1	0.71064	0.13958	25.921	<.0001	2.035	1.548	2.676
Urbanizaiton level 2 to 1	1	0.25279	0.14059	3.2328	0.0722	1.288	0.977	1.696
Urbanizaiton level 3 to 1	1	0.17116	0.19872	0.7418	0.3891	1.187	0.804	1.752
cci_1 to 0	1	1.32915	0.68917	3.7195	0.0538	3.778	0.979	14.584
cci_2 to0	1	1.43562	0.65872	4.7499	0.0293	4.202	1.156	15.282
cci_3 to 0	1	2.24838	0.61804	13.2344	0.0003	9.472	2.821	31.808

match Cox-regression

multi-Cox-regression

parameter	DF	estimate	StdErr	ChiSq	Pr > ChiSq	HazardRatio	HRUpper-HRLower CL	
group	1	0.35803	0.11558	9.5951	0.0020	1.431	1.141	1.794
Hypertension	1	0.55712	0.22912	5.9125	0.0150	1.746	1.114	2.735
Hyperlipidemia	1	0.01819	0.14754	0.0152	0.9019	1.018	0.763	1.36
Chronic kidney disease	1	-0.00757	0.14567	0.0027	0.9586	0.992	0.746	1.32
Depression	1	0.74953	0.15866	22.317	<.0001	2.116	1.55	2.888
Stroke	1	0.81386	0.14653	30.848	<.0001	2.257	1.693	3.007
Traumatic brain injury	1	0.49774	0.15378	10.4762	0.0012	1.645	1.217	2.224
Urbanizaiton level 2 to 1	1	0.20674	0.1564	1.7473	0.1862	1.230	0.905	1.671
Urbanizaiton level 3 t o1	1	0.12446	0.23613	0.2778	0.5981	1.133	0.713	1.799
cci_1 to 0	1	1.2335	0.7125	2.9971	0.0834	3.433	0.85	13.873
cci_2 to 0	1	1.03993	0.67562	2.3692	0.1238	2.829	0.753	10.635
cci_3 to 0	1	1.43665	0.65231	4.8505	0.0276	4.207	1.171	15.107

Match Cox-regression

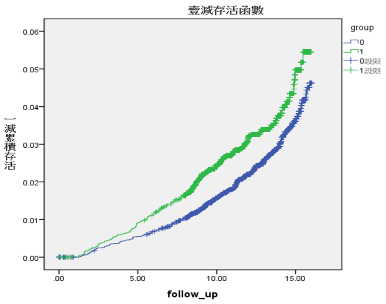

Supplement: Supplemental Information 1 [file peerj-05-3647-s001.pdf]
